# Supplementary material for: Xerotolerance: A New Property in Exiguobacterium Genus
Source: Microorganisms. 2021 Nov 28;9(12):2455. doi: 10.3390/microorganisms9122455 (PMC8706201; doi:10.3390/microorganisms9122455)
Supplement: Supplementary file 1 [file microorganisms-09-02455-s001.zip › Table S1.pdf]

Table S1. Plasmid pMCEX annotation at NCBI.

Locus	Length (pb)	Product	Protein id
HNY42_RS15710	144	hypothetical protein	WP_188005512.1
HNY42_RS16180	1698	hypothetical protein	WP_188005511.1
HNY42_RS16175	984	beta-ketoacyl-ACP synthase 3	WP_188005510.1
HNY42_RS16170	189	hypothetical protein	WP_188005509.1
HNY42_RS16165	804	SMI1/KNR4 family protein	WP_188005508.1
HNY42_RS16160	1035	hypothetical protein	WP_188005561.1
HNY42_RS16155	291	YolD-like family protein	WP_188005507.1
HNY42_RS16150	225	ribbon-helix-helix protein, CopG family	WP_188005506.1
HNY42_RS16145	270	type II toxin-antitoxin system RelE/ParE familytoxin	WP_188005505.1
HNY42_RS16140	720	HNH endonuclease	WP_188005504.1
HNY42_RS16135	612	hypothetical protein	WP_188005503.1
HNY42_RS16130	618	hypothetical protein	WP_188005502.1
HNY42_RS16125	4065	DNRLRE domain-containing protein	WP_188005501.1
HNY42_RS16120	273	hypothetical protein	WP_188005500.1
HNY42_RS16115	882	hypothetical protein	WP_188005499.1
HNY42_RS16110	1932	hypothetical protein	WP_188005498.1
HNY42_RS16105	1035	LD-carboxypeptidase	WP_188005497.1
HNY42_RS16100	1275	MFS transporter	WP_188005496.1
HNY42_RS16095	915	phosphotransferase	WP_188005495.1
HNY42_RS16090	1788	alanine--tRNA ligase	WP_188005494.1
HNY42_RS16085	642	deoxynucleoside kinase	WP_188005493.1
HNY42_RS16080	858	hypothetical protein	WP_188005492.1
HNY42_RS16075	774	hypothetical protein	WP_188005491.1
HNY42_RS16070	1227	hypothetical protein	WP_188005490.1
HNY42_RS16065	1200	ATP-grasp domain-containing protein	WP_188005489.1
HNY42_RS16060	1233	glycosyltransferase	WP_188005488.1
HNY42_RS16055	1071	asparaginase	WP_188005487.1
HNY42_RS16050	891	methyltransferase domain-containing protein	WP_188005486.1
HNY42_RS16045	1128	hypothetical protein	WP_188005485.1
HNY42_RS16040	960	NAD-dependent epimerase/dehydratase family protein	WP_188005484.1
HNY42_RS16035	1089	CDP-glucose 4,6-dehydratase	WP_188005483.1
HNY42_RS16030	801	NTP transferase domain-containing protein	WP_188005482.1
HNY42_RS16025	879	helix-turn-helix domain-containing protein	WP_188005481.1
HNY42_RS16020	477	hypothetical protein	WP_188005480.1
HNY42_RS16015	1935	AAA family ATPase	WP_188005479.1
HNY42_RS16010	1305	hypothetical protein	WP_188005478.1
HNY42_RS16005	798	DUF3800 domain-containing protein	WP_188005477.1
HNY42_RS16000	498	hypothetical protein	WP_188005476.1
HNY42_RS15995	651	hypothetical protein	WP_188005475.1
HNY42_RS15990	2502	hypothetical protein	WP_188005474.1
HNY42_RS15985	474	DNA mismatch endonuclease Vsr	WP_188005473.1
HNY42_RS15980	1269	DNA (cytosine-5-)-methyltransferase	WP_188005472.1
HNY42_RS15975	777	hypothetical protein	WP_188005471.1

HNY42_RS15970	231	hypothetical protein	WP_188005470.1
HNY42_RS15965	348	hypothetical protein	WP_188005560.1
HNY42_RS15960	171	hypothetical protein	WP_188005557.1
HNY42_RS15955	3354	hypothetical protein	WP_188005556.1
HNY42_RS15950	2574	helicase SNF2	WP_188005555.1
HNY42_RS15945	1215	hypothetical protein	WP_188005554.1
HNY42_RS15940	789	hypothetical protein	WP_188005553.1
HNY42_RS15935	777	AAA family ATPase	WP_188005552.1
HNY42_RS15930	690	ribbon-helix-helix protein, CopG family	WP_188005551.1
HNY42_RS15925	780	AAA family ATPase	WP_188005550.1
HNY42_RS15920	1077	hypothetical protein	WP_188005549.1
HNY42_RS15915	1437	hypothetical protein	WP_188005548.1
HNY42_RS15910	1476	hypothetical protein	WP_188005547.1
HNY42_RS15905	1155	hypothetical protein	WP_188005546.1
HNY42_RS15900	585	DUF4411 family protein	WP_188005545.1
HNY42_RS15895	507	helix-turn-helix domain-containing protein	WP_188005544.1
HNY42_RS15890	1017	tyrosine-type recombinase/integrase	WP_188005543.1
HNY42_RS15885	672	hypothetical protein	WP_188005542.1
HNY42_RS15880	981	hypothetical protein	WP_188005541.1
HNY42_RS15875	1182	hypothetical protein	WP_188005540.1
HNY42_RS15870	900	hypothetical protein	WP_188005539.1
HNY42_RS15865	489	helix-turn-helix domain-containing protein	WP_188005538.1
HNY42_RS15860	189	hypothetical protein	WP_188005537.1
HNY42_RS15855	225	hypothetical protein	WP_188005536.1
HNY42_RS15850	1092	hypothetical protein	WP_188005535.1
HNY42_RS15845	2370	type IV secretory system conjugative DNAtransfer family protein	WP_188005534.1
HNY42_RS15840	252	hypothetical protein	WP_119924055.1
HNY42_RS15835	429	hypothetical protein	WP_188005533.1
HNY42_RS15830	1311	hypothetical protein	WP_188005532.1
HNY42_RS15825	312	hypothetical protein	WP_188005531.1
HNY42_RS15820	636	hypothetical protein	WP_188005530.1
HNY42_RS15815	1944	type IV secretion system protein VirB4	WP_188005529.1
HNY42_RS15810	1065	lysozyme family protein	WP_188005559.1
HNY42_RS15805	558	hypothetical protein	WP_188005528.1
HNY42_RS15800	840	hypothetical protein	WP_188005527.1
HNY42_RS15795	147	hypothetical protein	WP_188005526.1
HNY42_RS15790	252	hypothetical protein	WP_188005525.1
HNY42_RS15785	1161	hypothetical protein	WP_188005524.1
HNY42_RS15780	456	hypothetical protein	WP_188005523.1
HNY42_RS15775	279	hypothetical protein	WP_188005522.1
HNY42_RS15770	1464	ImmA/IrrE family metallo-endopeptidase	WP_188005521.1
HNY42_RS15765	693	hypothetical protein	WP_188005520.1
HNY42_RS15760	162	hypothetical protein	WP_158537545.1
HNY42_RS15755	438	DUF5067 domain-containing protein	WP_188005558.1
HNY42_RS15750	225	DUF3006 family protein	WP_188005519.1
HNY42_RS15745	846	MBL fold metallo-hydrolase	WP_188005518.1

HNY42_RS15740	198	hypothetical protein	WP_188005517.1
HNY42_RS15735	861	S8 family serine peptidase	WP_131504374.1
HNY42_RS15730	915	hypothetical protein	WP_188005516.1
HNY42_RS15725	480	hypothetical protein	WP_188005515.1
HNY42_RS15720	387	GtrA family protein	WP_188005514.1
HNY42_RS15715	960	glycosyltransferase	WP_188005513.1
