# Supplementary material for: Xerotolerance: A New Property in Exiguobacterium Genus
Source: Microorganisms. 2021 Nov 28;9(12):2455. doi: 10.3390/microorganisms9122455 (PMC8706201; doi:10.3390/microorganisms9122455)
Supplement: Supplementary file 1 [file microorganisms-09-02455-s001.zip › Table S2.pdf]

Table S2. Genes from *Exiguobacterium* sp. Helios putatively involved in flagellum synthesis and functionality. The percentage of amino acids identity (%ID aa) is shown.

Gene	Locus	Function defined in B. subtilis subsp. subtilis str. 168	% ID aa	Accession No.
motA	*HNY42_RS00450*	Flagellar motor rotation protein	54.23%	P28611.1
motB	*HNY42_RS00455*	Flagellar motor rotation protein	45.87%	P28612.1
swrB	*HNY42_RS11055*	RNA polymerase sigma factor (σD) for flagellar operon	57.20%	P10726.2
cheD	*HNY42_RS11060*	Chemotaxis protein	45.16%	P40404.1
cheC	*HNY42_RS11065*	Chemotaxis protein, inhibitor of MCP methylation	34.50%	P40403.1
cheW	*HNY42_RS11070*	Positive regulator of CheA protein activity	40.88%	P39802.1
cheA	*HNY42_RS11075*	Signal transduction histidine kinase	48.36%	P29072.2
fleN	*HNY42_RS11080*	Flagellar synthesis regulator	36.08%	P40742.1
flhF	*HNY42_RS11085*	Flagellar biosynthesis protein	38.61%	Q01960.1
flhA	*HNY42_RS11090*	Flagellar biosynthesis protein	63.56%	P35620.2
flhB	*HNY42_RS11095*	Flagellar biosynthesis protein	50.28%	P35538.2
fliR	*HNY42_RS11100*	Flagellar biosynthesis protein	36.14%	P35537.2
fliQ	*HNY42_RS11105*	Flagellar biosynthesis protein	57.14%	P35535.1
fliP	*HNY42_RS11110*	Flagellar biosynthesis protein	64.65%	P35528.1
fliZ	*HNY42_RS11115*	Flagellar biosynthesis protein	29.47%	P35536.1
cheY	*HNY42_RS11120*	Chemotaxis regulator, transmits chemoreceptor signals to flagellar motor components.	77.50%	P24072.3
fliN	*HNY42_RS11125*	Flagellar motor switch protein	53.77%	P24073.2
fliM	*HNY42_RS11130*	Flagellar motor switch protein	53.44%	P23453.1
fliL	*HNY42_RS11135*	Flagellar biosynthesis protein	ND	
flbD	*HNY42_RS11140*	Flagellar protein	39.39%	C0H412.1
flgE	*HNY42_RS11145*	Flagellar basal-body rod protein	51.84%	P23446.2
flgD	*HNY42_RS11150*	Flagellar basal-body rod modification protein	44.88%	P23455.1
fliK	*HNY42_RS11155*	Flagellar hook-length control protein	ND	
flbB	*HNY42_RS11160*	Flagellar protein	ND	
fliJ	*HNY42_RS11165*	Flagellar protein	ND	
fliI	*HNY42_RS11170*	Flagellum-specific ATP synthase	63.49%	P23445.2
fliH	*HNY42_RS11175*	Flagellar assembly protein	ND	
fliG	*HNY42_RS11180*	Flagellar motor switch protein	71.00%	P23448.1
fliF	*HNY42_RS11185*	Flagellar M-ring protein	41.30%	P23447.2
fliE	*HNY42_RS11190*	Flagellar hook-basal body complex protein	39.81%	P24502.1
flgC	*HNY42_RS11195*	Flagellar basal-body rod protein	52.00%	P24501.1
flgB	*HNY42_RS11200*	Flagellar basal-body rod protein	40.34%	P24500.1
fliS	*HNY42_RS13935*	Flagellar secretion chaperone	ND	
	HNY42_RS13970		50.83%	P39739.1
fliD	*HNY42_RS13975*	Flagellar hook-associated protein	33.21%	P39738.2
yvyC	*HNY42_RS13980*	Flagellar protein	46.03%	P39737.1
flaA/hag	*HNY42_RS13940*	Flagellin protein	48.23%	P02968.2
	HNY42_RS13985		57.24%	
csrA	*HNY42_RS14055*	Translational regulator CsrA	63.01%	P33911.1
fliW	*HNY42_RS14060*	Flagellar assembly factor	40.29%	P96503.1
yviE	*HNY42_RS14065*	Uncharacterized protein	33.88%	P96502.1
flgL	*HNY42_RS14070*	Flagellar hook-associated protein	36.76%	P96501.1
flgK	*HNY42_RS14075*	Flagellar hook-associated protein	40.84%	P39810.2
flgN	*HNY42_RS14080*	Flagellar biosynthesis/type III secretory pathway chaperone	ND	
flgM	*HNY42_RS14085*	Anti-sigma-28 factor	ND	
flhP	*HNY42_RS15010*	Flagellar basal-body rod protein	32.23%	P39753.3
flhO	*HNY42_RS15015*	Flagellar basal-body rod protein	39.57%	P39752.2
