# Supplementary material for: Xerotolerance: A New Property in Exiguobacterium Genus
Source: Microorganisms. 2021 Nov 28;9(12):2455. doi: 10.3390/microorganisms9122455 (PMC8706201; doi:10.3390/microorganisms9122455)
Supplement: Supplementary file 1 [file microorganisms-09-02455-s001.zip › Table S3.pdf]

Table S3. *Exiguobacterium* sp. Helios genes that are not present in *E. sibiricum*

Locus	Protein
HNY42_RS00240	phosphotransferase
HNY42_RS01115	hypothetical protein
HNY42_RS01120	hypothetical protein
HNY42_RS01125	VOC family protein
HNY42_RS01140	DUF4230 domain-containing protein
HNY42_RS01145	hypothetical protein
HNY42_RS02265	phosphotransferase
HNY42_RS02920	hypothetical protein
HNY42_RS02930	copper resistance protein CopC
HNY42_RS03025	DUF4352 domain-containing protein
HNY42_RS03030	hypothetical protein
HNY42_RS03035	hypothetical protein
HNY42_RS03040	hypothetical protein
HNY42_RS03045	dynamin family protein
HNY42_RS03065	DEAD/DEAH box helicase family protein
HNY42_RS03070	TIR domain-containing protein
HNY42_RS03080	DUF3006 family protein
HNY42_RS03090	DUF262 domain-containing protein
HNY42_RS03095	hypothetical protein
HNY42_RS03100	hypothetical protein
HNY42_RS03115	extracellular solute-binding protein
HNY42_RS03155	excalibur calcium-binding domain-containing protein
HNY42_RS03175	class I SAM-dependent methyltransferase
HNY42_RS03970	IS3 family transposase
HNY42_RS06980	hypothetical protein
HNY42_RS07210	helix-turn-helix domain-containing protein
HNY42_RS07235	hypothetical protein
HNY42_RS07240	MepB family protein
HNY42_RS07245	RNA polymerase sigma-70 factor
HNY42_RS07255	MarR family transcriptional regulator
HNY42_RS07260	NtaA/DmoA family FMN-dependent monooxygenase
HNY42_RS07270	NAD(P)-binding domain-containing protein
HNY42_RS07465	antibiotic biosynthesis monooxygenase
HNY42_RS07490	hypothetical protein
HNY42_RS07495	hypothetical protein
HNY42_RS07505	hypothetical protein
HNY42_RS07510	Petrobactin biosynthesis protein AsbE
HNY42_RS07515	TIM barrel protein
HNY42_RS07545	hypothetical protein
HNY42_RS07680	spore coat protein
HNY42_RS07685	spore coat protein
HNY42_RS07695	DUF2817 domain-containing protein
HNY42_RS07740	hypothetical protein
HNY42_RS07820	GNAT family N-acetyltransferase
HNY42_RS07825	SMI1/KNR4 family protein
HNY42_RS07830	hypothetical protein
HNY42_RS07845	hypothetical protein
HNY42_RS07950	hypothetical protein
HNY42_RS07975	PTS sugar transporter subunit IIB
HNY42_RS07980	PTS lactose/cellobiose transporter subunit IIA
HNY42_RS07985	PTS cellobiose transporter subunit IIC
HNY42_RS08000	DUF624 domain-containing protein

HNY42_RS08010	sugar ABC transporter substrate-binding protein
HNY42_RS08025	family 43 glycosylhydrolase
HNY42_RS08035	L-ribulose-5-phosphate 4-epimerase
HNY42_RS08040	L-arabinose isomerase
HNY42_RS08045	alpha-N-arabinofuranosidase
HNY42_RS08055	iron-containing alcohol dehydrogenase
HNY42_RS08060	family 43 glycosylhydrolase
HNY42_RS08065	family 43 glycosylhydrolase
HNY42_RS08070	hypothetical protein
HNY42_RS08080	hypothetical protein
HNY42_RS08120	hypothetical protein
HNY42_RS08165	helix-turn-helix domain-containing protein
HNY42_RS08190	hypothetical protein
HNY42_RS08200	barstar family protein
HNY42_RS08215	cupin
HNY42_RS08230	WYL domain-containing protein
HNY42_RS08300	DUF4304 domain-containing protein
HNY42_RS08305	hypothetical protein
HNY42_RS08410	class I SAM-dependent methyltransferase
HNY42_RS08415	hypothetical protein
HNY42_RS08440	acetoacetate decarboxylase family protein
HNY42_RS08715	hypothetical protein
HNY42_RS08720	GNAT family N-acetyltransferase
HNY42_RS08735	DUF1048 domain-containing protein
HNY42_RS08740	helix-turn-helix transcriptional regulator
HNY42_RS08745	CPBP family intramembrane metalloprotease
HNY42_RS08810	chitin disaccharide deacetylase
HNY42_RS08820	6-phospho-beta-glucosidase
HNY42_RS08825	PTS transporter subunit EIIC
HNY42_RS08830	PTS lactose/cellobiose transporter subunit IIA
HNY42_RS08835	PTS sugar transporter subunit IIB
HNY42_RS09120	hypothetical protein
HNY42_RS09125	ABC transporter permease subunit
HNY42_RS09185	MarR family transcriptional regulator
HNY42_RS09190	hypothetical protein
HNY42_RS09230	DUF3169 family protein
HNY42_RS09275	ABC transporter permease
HNY42_RS09280	hypothetical protein
HNY42_RS09305	IS3 family transposase
HNY42_RS09340	MarR family transcriptional regulator
HNY42_RS09450	phosphotransferase
HNY42_RS09455	phosphotransferase
HNY42_RS09530	group-specific protein
HNY42_RS09570	serine hydrolase
HNY42_RS12045	hypothetical protein
HNY42_RS13520	DUF2785 domain-containing protein
HNY42_RS13615	AAA family ATPase
HNY42_RS13700	TIR domain-containing protein
HNY42_RS13705	hypothetical protein
HNY42_RS13710	hypothetical protein
HNY42_RS13720	hypothetical protein
HNY42_RS13725	hypothetical protein
HNY42_RS14690	hypothetical protein
HNY42_RS14745	DUF4163 domain-containing protein

HNY42_RS14755	hypothetical protein
HNY42_RS14785	hypothetical protein
HNY42_RS14790	hypothetical protein
HNY42_RS14795	DUF3267 domain-containing protein
HNY42_RS14800	oligosaccharide repeat unit polymerase
HNY42_RS14810	hypothetical protein
HNY42_RS14815	glycosyltransferase family 4 protein
HNY42_RS14830	NAD-dependent epimerase/dehydratase family protein
HNY42_RS15485	alpha/beta fold hydrolase
HNY42_RS15490	TetR family transcriptional regulator
HNY42_RS15510	hypothetical protein
HNY42_RS15515	AAA family ATPase
HNY42_RS15540	DUF2089 family protein
HNY42_RS15545	hypothetical protein
