# Supplementary material for: Xerotolerance: A New Property in Exiguobacterium Genus
Source: Microorganisms. 2021 Nov 28;9(12):2455. doi: 10.3390/microorganisms9122455 (PMC8706201; doi:10.3390/microorganisms9122455)
Supplement: Supplementary file 1 [file microorganisms-09-02455-s001.zip › Table S4.pdf]

Table S4. *E. sibiricum* genes that are not present in *Exiguobacterium* sp. Helios

Locus	Protein
EXIG_RS00960	hypothetical protein
EXIG_RS00985	CRISPR-associated helicase/endonuclease Cas3
EXIG_RS00990	type I-C CRISPR-associated protein Cas5
EXIG_RS00995	type I-C CRISPR-associated protein Cas8c/Csd1
EXIG_RS01000	type I-C CRISPR-associated protein Cas7/Csd2
EXIG_RS01005	CRISPR-associated protein Cas4
EXIG_RS01010	type I-C CRISPR-associated endonuclease Cas1
EXIG_RS01015	CRISPR-associated endonuclease Cas2
EXIG_RS01470	IS1182 family transposase
EXIG_RS01520	IS30 family transposase
EXIG_RS01685	hypothetical protein
EXIG_RS01695	GHKL domain-containing protein
EXIG_RS01795	hypothetical protein
EXIG_RS01800	DUF1819 family protein
EXIG_RS01805	DUF1788 domain-containing protein
EXIG_RS01810	BREX system P-loop protein BrxC
EXIG_RS01815	BREX-1 system adenine-specific DNA-methyltransferase PglX
EXIG_RS01820	BREX-1 system phosphatase PglZ type A
EXIG_RS01825	protease Lon-related BREX system protein BrxL
EXIG_RS01830	AAA family ATPase
EXIG_RS01835	HNH endonuclease
EXIG_RS01875	IS30 family transposase
EXIG_RS01940	hypothetical protein
EXIG_RS02790	IS30 family transposase
EXIG_RS04040	IS200/IS605 family transposase
EXIG_RS04535	ferrous iron transport protein A
EXIG_RS04540	ferrous iron transport protein B
EXIG_RS04545	FeoB-associated Cys-rich membrane protein
EXIG_RS05245	IS30 family transposase
EXIG_RS05930	IS30 family transposase
EXIG_RS05965	IS30 family transposase
EXIG_RS05970	N-acetylmuramoyl-L-alanine amidase
EXIG_RS06015	nucleotidyltransferase domain-containing protein
EXIG_RS06020	hypothetical protein
EXIG_RS06130	IS200/IS605 family transposase
EXIG_RS06135	transposase
EXIG_RS06225	IS1182 family transposase
EXIG_RS06595	hypothetical protein
EXIG_RS06600	hypothetical protein
EXIG_RS06605	hypothetical protein
EXIG_RS06610	ABC transporter permease
EXIG_RS06630	DMT family transporter
EXIG_RS06635	hypothetical protein
EXIG_RS06700	geobacillin-26 family protein
EXIG_RS06705	transposase
EXIG_RS06745	suppressor of fused domain protein
EXIG_RS06865	hypothetical protein
EXIG_RS06895	hypothetical protein
EXIG_RS06900	hypothetical protein
EXIG_RS06905	LysE family transporter
EXIG_RS06915	AraC family transcriptional regulator
EXIG_RS06930	dihydrodipicolinate synthase family protein

EXIG_RS06970	hypothetical protein
EXIG_RS06980	hypothetical protein
EXIG_RS07035	hypothetical protein
EXIG_RS07085	NAD(P)H-dependent oxidoreductase
EXIG_RS07090	MarR family transcriptional regulator
EXIG_RS07230	HNH endonuclease
EXIG_RS07240	hypothetical protein
EXIG_RS07310	peptidase E
EXIG_RS07320	NUDIX domain-containing protein
EXIG_RS07325	Fic family protein
EXIG_RS07375	bacteriorhodopsin
EXIG_RS07410	hypothetical protein
EXIG_RS07510	YnfA family protein
EXIG_RS07715	hypothetical protein
EXIG_RS07775	IS1182 family transposase
EXIG_RS07800	hypothetical protein
EXIG_RS07920	hypothetical protein
EXIG_RS08065	alkaline shock response membrane anchor protein AmaP
EXIG_RS08070	DUF2273 domain-containing protein
EXIG_RS08075	Asp23/Gls24 family envelope stress response protein
EXIG_RS08435	IS200/IS605 family transposase
EXIG_RS10535	IS30 family transposase
EXIG_RS10545	IS30 family transposase
EXIG_RS11140	ABC transporter permease
EXIG_RS11355	IS30 family transposase
EXIG_RS11865	IS1182 family transposase
EXIG_RS12100	site-specific DNA-methyltransferase
EXIG_RS12105	helix-turn-helix transcriptional regulator
EXIG_RS12110	restriction endonuclease BglII
EXIG_RS12115	AIPR family protein
EXIG_RS12750	hypothetical protein
EXIG_RS12755	hypothetical protein
EXIG_RS13215	ImmA/IrrE family metallo-endopeptidase
EXIG_RS13220	hypothetical protein
EXIG_RS13230	hexose-6-phosphate:phosphate antiporter
EXIG_RS13235	extracellular solute-binding protein
EXIG_RS13240	DUF3919 family protein
EXIG_RS13255	DUF5011 domain-containing protein
EXIG_RS13270	hypothetical protein
EXIG_RS13275	hypothetical protein
EXIG_RS13280	zinc ribbon domain-containing protein
EXIG_RS13285	hypothetical protein
EXIG_RS13290	zinc ribbon domain-containing protein
EXIG_RS13295	zinc ribbon domain-containing protein
EXIG_RS13305	hypothetical protein
EXIG_RS13330	hypothetical protein
EXIG_RS13345	oligosaccharide flippase family protein
EXIG_RS13350	hypothetical protein
EXIG_RS13450	IS30 family transposase
EXIG_RS13715	ATP-binding protein
EXIG_RS13985	hypothetical protein
EXIG_RS14010	hypothetical protein
EXIG_RS14020	HEAT repeat domain-containing protein
EXIG_RS14030	hypothetical protein

EXIG_RS14900	FAD:protein FMN transferase
EXIG_RS14905	hypothetical protein
EXIG_RS14960	IS1182 family transposase
EXIG_RS15210	hypothetical protein
EXIG_RS15335	hypothetical protein
EXIG_RS15340	hypothetical protein
EXIG_RS15585	hypothetical protein
EXIG_RS15635	hypothetical protein
EXIG_RS15675	IS3 family transposase
EXIG_RS15685	IS3 family transposase
EXIG_RS15710	IS30 family transposase
EXIG_RS15715	type II toxin-antitoxin system mRNA interferase toxin, RelE/StbE family
EXIG_RS15730	IS3 family transposase
EXIG_RS15740	transposase
EXIG_RS15745	IS3 family transposase
EXIG_RS15775	IS3 family transposase
EXIG_RS15905	hypothetical protein
EXIG_RS15915	hypothetical protein
EXIG_RS15925	hypothetical protein
EXIG_RS15945	hypothetical protein
EXIG_RS15960	hypothetical protein
EXIG_RS15965	hypothetical protein
EXIG_RS15970	hypothetical protein
