# Supplementary material for: Xerotolerance: A New Property in Exiguobacterium Genus
Source: Microorganisms. 2021 Nov 28;9(12):2455. doi: 10.3390/microorganisms9122455 (PMC8706201; doi:10.3390/microorganisms9122455)
Supplement: Supplementary file 1 [file microorganisms-09-02455-s001.zip › Table S5.pdf]

Table S5. Other strains isolated by desiccation assay.

Phylum	Class	Order	Family	Genus	Strain	Closest type strain
Proteobacteria	Alphaproteobacteria	Caulobacteriales	*Caulobacteriaceae*	*Brevundimonas*	HE24.1	*Brevundimonas vesicularis*
Firmicutes	Bacilli	Bacillales	*Incertae sedis*	*Exiguobacterium*	HE26.4	*Exiguobacterium mexicanum*
			Bacillaceae	*Bacillus*	HE19.6	*Bacillus pumilus*
				Bacillus	H19.15	*Bacillus megaterium*
				Bacillus	HE20.1	*Bacillus licheniformis*
				Bacillus	HE20.17	*Bacillus licheniformis*
				Bacillus	HE24.8	*Bacillus licheniformis*
				Bacillus	HE24.9	*Bacillus licheniformis*
				Bacillus	HE23.1	*Bacillus paralicheniformis*
				Bacillus	HE24.11	*Bacillus megaterium*
				Bacillus	HE20.18	*Bacillus safensis*
				Bacillus	HE20.19	*Bacillus safensis*
				Bacillus	HE24.15	*Bacillus circulans*
				Bacillus	HE21.16	*Bacillus taxi*
			Paenibacillaceae	*Paenibacillus*	HE24.5	*Paenibacillus xylanilyticus*
				Paenibacillus	HE24.6	*Paenibacillus xylanilyticus*
				Paenibacillus	HE24.7	*Paenibacillus xylanilyticus*
				Paenibacillus	HE24.13	*Paenibacillus xylanilyticus*
				Paenibacillus	E2A	*Paenibacillus xylanilyticus*
				Paenibacillus	E2B	*Paenibacillus xylanilyticus*
				Paenibacillus	E5	*Paenibacillus lautus*
			Planococcaceae	*Planococcus*	HE26.1	*Planococcus donghaensis*
				Planococcus	HE25.5Y	*Planococcus donghaensis*
				Planococcus	HE21.14	*Planococcus donghaensis*
				Planococcus	HE26.2 (secA)	*Planococcus glaciei*
				Planococcus	HE26.2 (secB)	*Planococcus glaciei*
				Planococcus	HE25.5H	*Planococcus okeanokoikes*
Actinobacteria	Actinomycetia	Micrococcales	*Micrococcaceae*	*Arthrobacter*	HE24.10	*Arthrobacter luteolus*
				Arthrobacter	HE26.12	*Arthrobacter gandavensis*

				Arthrobacter	HE26.26	*Arthrobacter bussei*
				Paenarthrobacter	HE19.5	*Paenarthrobacter nitroguajacolicus*
				Pseudoarthrobacter	HE19.16	*Pseudoarthrobacter oxydans*
				Micrococcus	HE26.32A	*Micrococcus luteus*
				Kokuria	HE26.7A	*Kocuria gwangalliensis*
				Kokuria	HE26.24A	*Kocuria gwangalliensis*
				Kokuria	HE26.6	*Kocuria gwangalliensis*
				Kokuria	HE26.30B	*Kocuria rosea*
Actinobacteria	Actinomycetia	Mycobacteriales	*Nocardiaceae*	*Rhodococcus*	HE24.12	*Rhodococcus ruber*
				Rhodococcus	HE24.14	*Rhodococcus coprophilus*
			Gordoniaceae	*Gordonia*	HE24.3	*Gordonia terrae*
				Gordonia	HE24.4J	*Gordonia terrae*
		Cellulomonadales	*Promicromonosporaceae*	*Cellulosimicrobium*	HE19.14	*Cellulosimicrobium Funkei*
				Krasilnikoviella	HE20.9	*Krasilnikoviella muralis*
		Streptosporangiales	*Nocardiopsaceae*	*Nocardiopsis*	HE24.2	*Nocardiopsis umidischolae*
		Microbacteriales	*Microbacteriaceae*	*Microbacterium*	HE21.26	*Microbacterium esteraromaticum*
				Microbacterium	HE21.24	*Microbacterium paraoxydans*
				Microbacterium	HE21.32	*Microbacterium esteraromaticum*
				Microbacterium	HE21.37	*Microbacterium thalassium*
