# Supplementary figures and images for: Xerotolerance: A New Property in Exiguobacterium Genus
Source: Microorganisms. 2021 Nov 28;9(12):2455. doi: 10.3390/microorganisms9122455 (PMC8706201; doi:10.3390/microorganisms9122455)

Figure S1

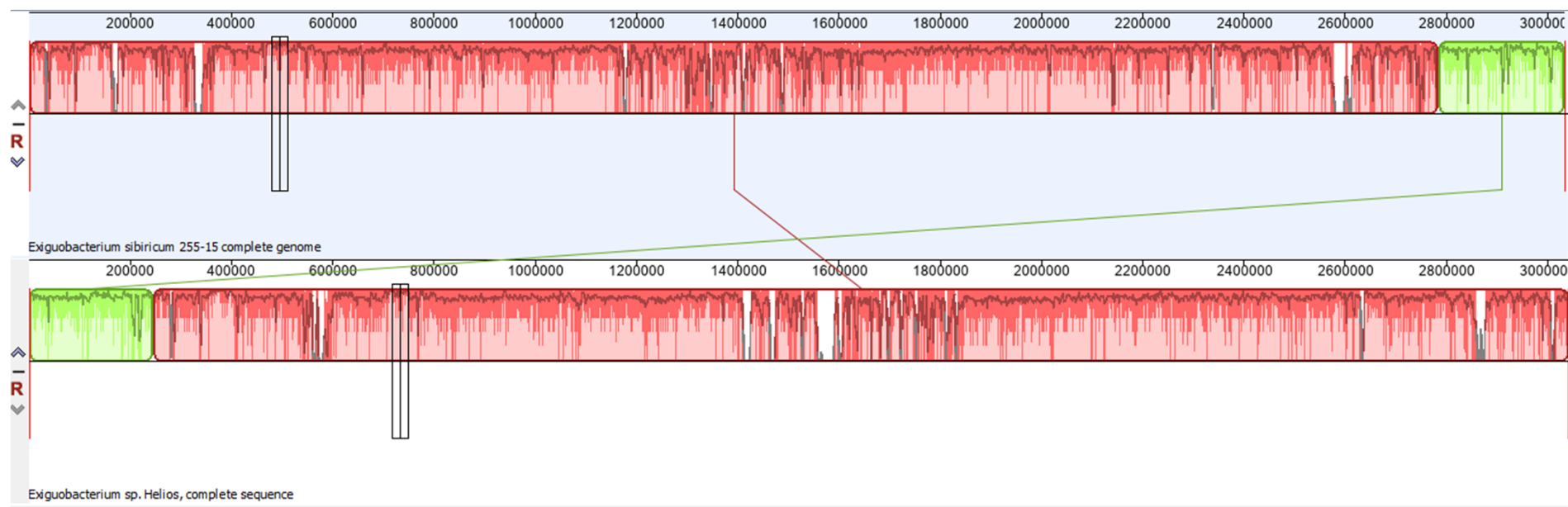

Figure S2

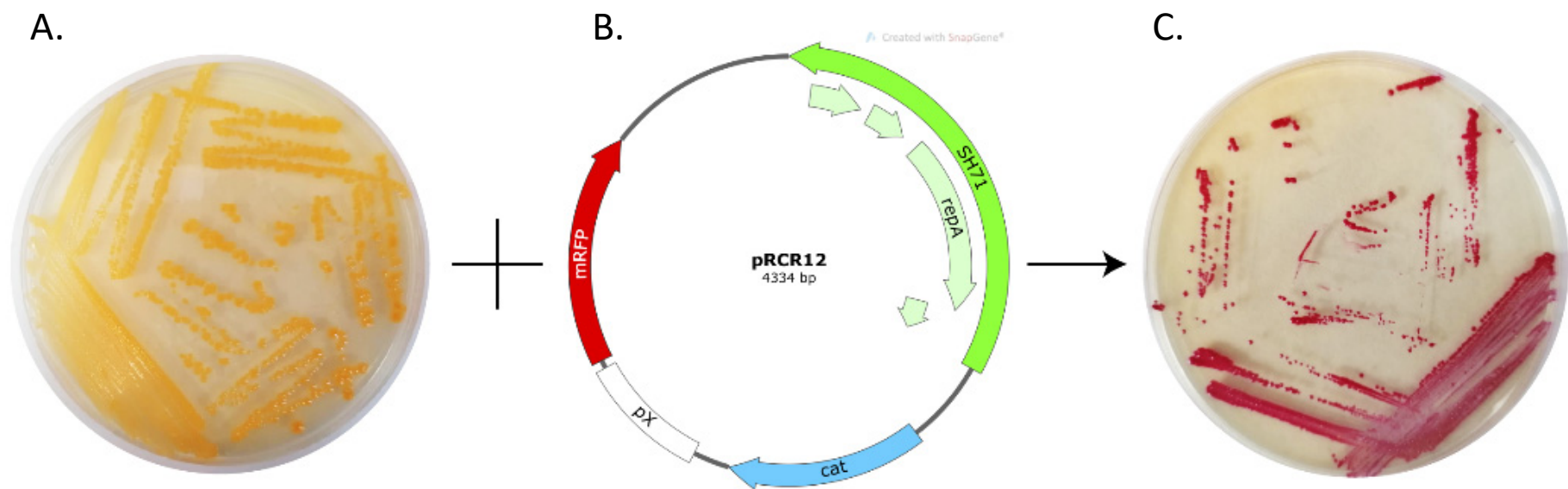

Figure S3

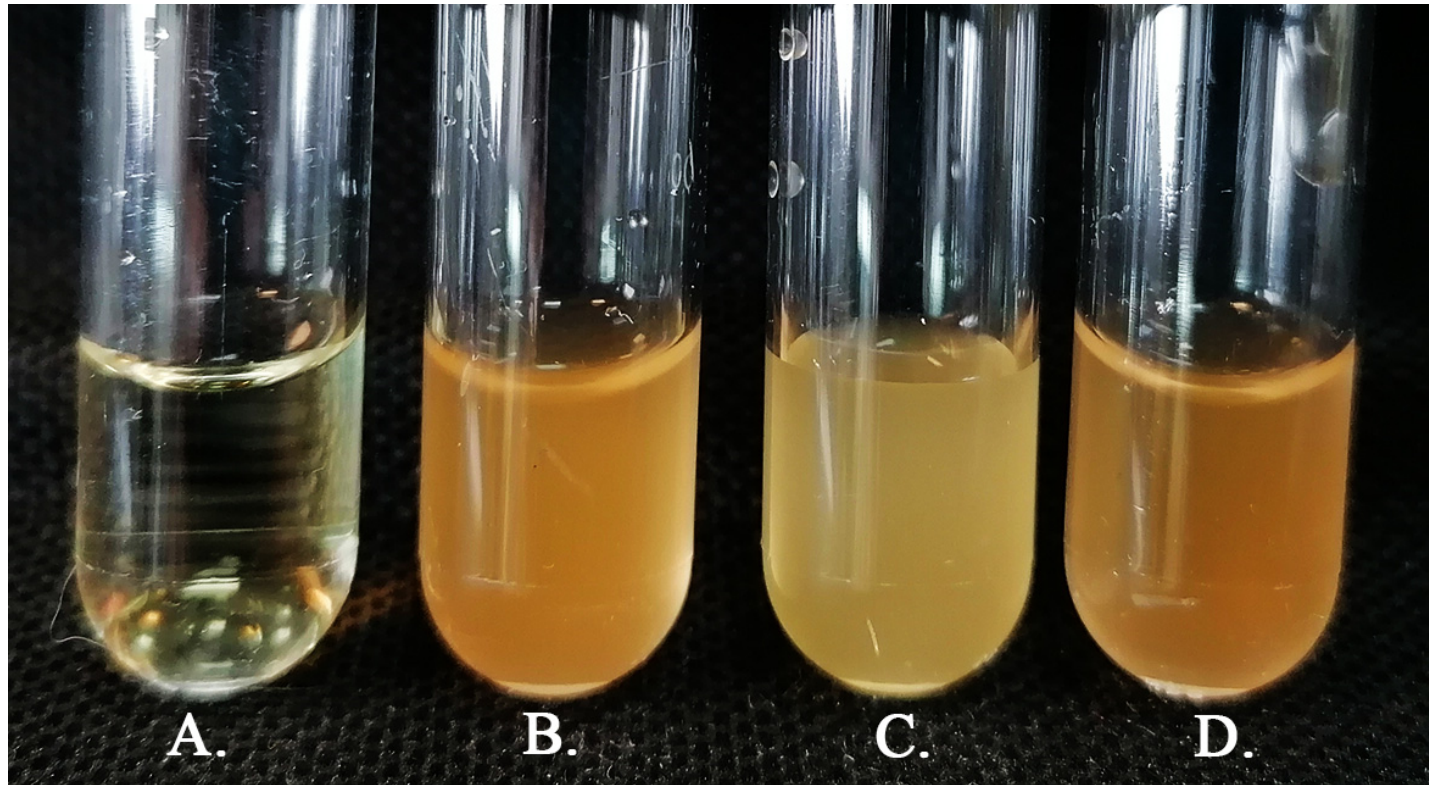

Supplement: Supplementary file 1 [file microorganisms-09-02455-s001.zip › Supplementary figures.pdf]
